# A novel multistage ensemble approach for prediction and classification of diabetes

**DOI:** 10.3389/fphys.2022.1085240

**Published:** 2022-12-19

**Authors:** Sarita Simaiya, Rajwinder Kaur, Jasminder Kaur Sandhu, Majed Alsafyani, Roobaea Alroobaea, Deema mohammed alsekait, Martin Margala, Prasun Chakrabarti

**Affiliations:** ^1^ Department of Computer Science and Engineering, Institute of Engineering and Technology, Chandigarh University, Mohali, Punjab, India; ^2^ School of Computing and Informatics, University of Louisiana, Lafayette, LA, United States; ^3^ Chitkara University Institute of Engineering and Technology, Chitkara University, Rajpura, Punjab, India; ^4^ Department Science, College of Computers and Information Technology, Taif University, Taif, Saudi Arabia; ^5^ Department of Computer Science, College of Computers and Information Technology, Taif University, Taif, Saudi Arabia; ^6^ Department of Computer Science and Information Technology, Applied College, Princess Nourah bint Abdulrahman University, Riyadh, Saudi Arabia; ^7^ ITM (SLS) Baroda University, Vadodara, Gujarat, India

**Keywords:** health care, diabetes mellitus, clinical data processing, ensemble modeling, machine learning, prediction, linear discriminant analysis

## Abstract

Diabetes mellitus is a metabolic syndrome affecting millions of people worldwide. Every year, the rate of occurrence rises drastically. Diabetes-related problems across several vital organs of the body can be fatal if left untreated. Diabetes must be detected early to receive proper treatment, preventing the condition from escalating to severe problems. Tremendous health sciences and biotechnology advancements have resulted in massive data that generated massive Electronic Health Records and clinical information. The exponential increase of electronically gathered information has resulted in more complicated, accurate prediction models that can be updated continuously using machine learning techniques. This research mainly emphasizes discovering the best ensemble model for predicting diabetes. A new multistage ensemble model is proposed for diabetes prediction. In this model, accuracy is predicated on the Pima Indian Diabetes dataset. The accuracy of the proposed ensemble model is compared with the existing machine learning model, and the experimental results demonstrate the performance of the proposed model in terms of higher Precision, f-measure, Recall, and area under the curve.

## 1 Introduction

Diabetes Mellitus (DM) is a common chronic endocrine disorder spread worldwide. This disease will affect 463 million people worldwide by 2019 and will increase to 629 million by 2045 (Ahmad et al., 2021). According to the International Diabetes Federation (IDF) report, Middle East countries are more prone to DM. World Health Organization (WHO) declares Saudi Arabia country considers fifth rank in high diabetes. It is assumed that if the high incidence rate of DM is not controlled timely in Saudi Arabia, it will take the first rank in diabetes by 2035 (Akram et al., 2014). This uncontrolled DM burdens government health expenses, approximately 17 billion Riyals. DM syndrome affects the body’s metabolism and can cause structural changes. The reason for this syndrome is an insufficient amount of insulin-making in the body, which leads to distinct health issues such as heart disease, premature death, kidney failure, blindness, stroke, and lower limb amputation (Al-Rubeaan et al., 2014). It is a lifestyle disease that results in obesity because of physical inactivity.

DM is categorized into three types: Type1, Type2, and gestational (Alsuliman et al., 2021). In Type 1 DM, little insulin is released in the body due to the immune system’s attack on pancreatic beta cells (Antal and Hajdu, 2014). Type 2 is more dangerous than type 1 and can cause death. In this case, the body either does not produce insulin or becomes insulin resistant (Araya et al., 2021). Gestational DM arises only during pregnancy because of hormonal changes in the body. Prediction of DM stimulates great interest in itself and becomes a demanding issue in health monitoring. Due to the rapid growth of this syndrome, researchers emphasize the country’s DM status, frequently determine people’s self-care behavior (Bertachi et al., 2020), and identify diabetes control factors and the role of Machine Learning (ML) models in predicting DM (Çalişir and Doǧantekin, 2011). These efforts of researchers help to enhance the quality of the healthcare sector. It also improves the accuracy rate of predicting diabetes in the early stages. Diabetes prediction can be measured on several criteria. Some are unproductive such as WBC Count, fructose amine, logical hematoma index, and fibrinogen. The predictive analysis mainly shows the significance of DM. It uses ML models to estimate indefinite results. It comprises diagnosis, prediction, self-management, and prevention.

Various ML approaches have been applied to classify and predict accurate diabetes, such as Logistic Regression (LR), SVM, Naïve Bayes (NB), Random Forest (RF) and Decision Tree (DT), Neural networks (NN), Extreme Learning Machine (ELM). All approaches have their advantages and disadvantages. Most of them are restricted to the benefits of a single concept. In many kinds of research, heuristics, techniques, and computational intelligence was also applied to predict DM. However, no single classifier performs best in predicting diabetes. This problem can be resolved by using the Ensemble ML approach (de Bruijne, 2016; Filippatos et al., 2021). It is an approach in which two or more ML algorithms are combined to increase the limit of the single ML algorithm. The ensemble ML framework is dependent on the research problem. In comparison to a single algorithm, it produces more accurate results. Due to urbanization, the lifestyle of the people changes and increases the cases of diabetics. It becomes life-threatening when its associated symptoms are not visible and remains undiagnosed for several years. So, it is necessary to classify and predict DM to minimize the disease’s occurrence and complications during the early stages. The various ML approaches are applied to predict accurate diagnosis in which ensemble ML provides the best results.

This research proposes a new multistage ensemble model to predict diabetes with accuracy on the Pima Indian Diabetes (PID) dataset. Six ML techniques are used in the proposed ensemble approach. Naïve Bayes, KNN and J48 are used in stage I ensemble. Random Forest and JRip are used in the stage II ensembles. The output of stage I and stage II are input in stage III. SVM is a good classifier used at the last stage III ensemble to predict diabetic disease. It gives a generalized performance and does not produce complexity when learning with all combinations of features. The characteristics of SVM play a significant role in the prediction of DM. This ensemble approach is provided using the python tool. It gives the best results regarding Recall, Precision, F-Measure, and AUC values. The complete research paper is organized as follows: Section 2 discusses the existing work analysis on DM for the past few years. Section 3 elaborates on the materials and methods. Section 4 discusses the experimental results and discussion and compares the proposed approach with existing approaches. Section 5 concludes the research article.

## 2 Existing work

In the medical field, image recognition and feature selection techniques are prevalent among researchers. Based on prime interest to enhance the physical examination facility for patients and doctors and provide the necessary data for examining essential health. In this paper, we cover diabetes and its types. How to detect diabetes accurately? In the survey work, various techniques are discussed related to the research. Researchers have applied different ML approaches to classify and predict diabetes, including LR, SVM, NB, RF, and DT. These are all the methods we have covered in the literature work. The macula is a type of diabetes, to detect the exact pattern of the macula with the help of the Gaussian model (Garcia-Carretero et al., 2020). The researcher proposed a hybrid approach, including the Gaussian and SVM methods, to recognize the feature even in other bright lesions, eventually leading to the reliable classification of input retinal images in different stages of macular edema. Two databases, HEI-MED and MESSIDOR, have been used in this work. One hundred sixty-nine images and 1,200 high-quality images from the first database were used for feature selection from the second database. The existing and proposed systems’ performance was compared based on various performance measuring parameters. The experimental results show a better version of the proposed model.

Similarly, in the paper (Harimoorthy and Thangavelu, 2020), the author developed a hybrid approach for recognizing diabetic retinopathy (DR). These techniques extracted patterns from the images, i.e., quality assessment, pre-screening, AM/FM, *etc.* The experimental results have shown a better performance of the proposed model. In the medical field, a considerable number of datasets are available. In the paper (Hasan Mahmud et al., 2018), Pima Indian Diabetes collected from the UCI repository is used for finding the relationship between healthcare resources and the challenges sector. In this work, various ML techniques, i.e., K-Means and SVM, were used to detect diabetes. The outcome of the proposed SVM classifier produces 96.04% accuracy compared to existing methods. The complex problem cannot be easily identified and cured due to some environmental factors and lifestyles proven by many medical researchers. However, the existing research has shown no standard approach to detecting the disease. The author proposed a novel approach to provide the best way to detect complex diseases like type-2 DM (Islam and Jahan, 2017). A dynamic weighted voting scheme called multiple factors weighted combination (MFWC) is discussed in this paper. Here, kNN and SVM ML techniques have been used for classification. WEKA tools were used for implementation. The experimental results of the research have shown better results than existing methods for diabetes detection. However, the proposed method could not identify type-2 diabetes at some levels.

In the article (Islam et al., 2020a), researchers emphasize the country’s DM status, frequently determine people’s self-care behavior, and identify diabetes control factors and the role of ML models in predicting DM. The main focus was on the detection of type-2 diabetes. In this research, 106 attributes and 844 instances were used. Dataset is collected from the Indian people under surveillance of medical. The k-means clustering approach was used to detect type-2 DM. The proposed model is not fit to see blood sugar %. A neural networks-based model has been utilized in (Islam et al., 2020b) to predict the onset of diabetes mellitus on the Prima Indian Diabetes dataset. They showed that their approach is reliable for such classification. This study aims to detect the diabetic patient’s onset from the outcomes generated by the machine learning classification algorithm. SVM, NB, and logical regression techniques are used. The 10- fold cross Metris technique was used. For better accuracy, various action analyses and function selection combinations are required. Extract the features from an average person and a diabetic person. However, the researcher (Kassahun et al., 2016) applied Machine Learning techniques to examine diabetic and ordinary people. They determine the data set that is related to real-time. Data sets are collected from Pima Indian population near Phoenix, Arizona. The WEKA tool builds a model to identify normal and diabetic patients. The authors trained the model using the RF, NB, and J48 methods. Precision, Recall, F-Measure, and ROC were utilized for evaluation metrics. The research (Krause et al., 2018) states that diabetes is a disease that increases daily. This paper uses the ML approaches, namely neural network, DT, and RF, to detect diabetes. The data set included in this paper is collected by physically examining the patents in Luzhou, China. The dataset consists of 14 columns. MATLAB and WEKA are used for simulation. As suggested, the system cannot find the type of diabetes and cannot explore the portion of each indicator. In research (Lai et al., 2019), the authors focus on designing a model that anonymous the prediction of diabetes in affected people with higher accuracy. The authors use the ML approaches to identify diabetes based on past data fed into a model. The ML techniques have used diabetes at an earlier stage. Pima data set is used, and the WEKA tool is used to analyze the result. DT, SVM, and NB machine learning techniques are used. However, few authors (Li et al., 2021a) use deep learning techniques for analysis. Applying the TFDA-1 Tongue Diagnosis Instrument (Li et al., 2021b), the authors collect tongue images and extract tongue features, including color and texture features, using TDAS.

In (Mercaldo et al., 2017), features can improve the prediction accuracy of the diabetes risk prediction model formed by the classical machine learning model significantly. They observed that the literature showed that detecting diabetes with the help of ML techniques is much easier (Periyasamy et al., 2011). The authors state that the proposed model is highly effective and provides better accuracy. They collected data from the Laboratory and other analytics results of Canadian Patients. Authors use the ML technique to predict the model. They only use the 13,309 patients’ records. This proposed model is further used for online system programs to help clinicians predict diabetes and give the essential techniques to handle it. Prediction (Polat et al., 2008) of Diabetes stimulates a great interest and becomes a demanding issue in health monitoring. The authors used data mining approaches to predict the risk factor. They used different ML approaches to predict diabetes. They collected data from interactions with patients and figured out the symptom of the disease. Weka tool is one of the tools that were used for analysis. Besides this 10-fold cross-validation technique is used for evaluation. The article’s main focus (Prasad et al., 2020) is to use the essential features instead of all attributes. Furthermore, researchers finalize the data. When preparing the data, they use different preparation methods to avoid outliers from the data set. The authors proposed the regression machine learning technique. However, in (Ram and Vishwakarma, 2021) author can only analyze of type of diabetes. Diabetes mellitus is a common chronic endocrine disorder spread all over the world. The authors find out the risks and focus on the calculation system. The authors developed a model based on eXtreme Gradient Boosting (XGBoost). To improve the accuracy and risk analysis, the ML technique is used. Researchers must build a system to guide human health management for better results. There is evidence (Ram and Vishwakarma, 2021) about a possible relationship between thyroid abnormalities and gestational diabetes mellitus (GDM). However, there is still no conclusive data on this dependence since no strong correlation has been proven.

### 2.1 Comparative study of various diabetes prediction methods

Table 1 represents the comparative analysis of various diabetes prediction Methods based on different parameters.

**TABLE 1:** Comparative study of various diabetes prediction Methods.

References	Classification of diabetes	Prediction of diabetes	Data set used	Key parameters used
Ram and Vishwakarma, (2021)	Yes	No	Kaggle Dataset	Precision, Recall, Accuracy
Somasundaram and Alli, (2017)	No	Yes	UCI Dataset	Accuracy
Saad et al. (2018)	No	Yes	Offline Dataset	Precision, Recall, F-Measure Accuracy
Saleh et al. (2018)	No	No	Offline Dataset	Accuracy
Santhanam and Padmavathi, (2015)	No	Yes	UCI Data set	Precision and Recall
Singh and Singh, (2020)	No	Yes	Kaggle Dataset	Precision and standard deviation
Sisodia and Sisodia, (2018)	No	No	Offline Dataset	Accuracy
Kumar Bhoi et al. (2021)	No	Yes	UCI Data set	Precision
Syed and Khan, (2020)	No	Yes	Kaggle Dataset	Precision
Worachartcheewan et al. (2015)	No	No	Kaggle Dataset	Precision and standard deviation
Yang et al. (2021)	No	Yes	Offline Dataset	Accuracy
Zhu et al. (2015)	No	Yes	UCI Data set	Precision
Zou et al. (2018)	No	No	Kaggle Dataset	Precision
Trivedi et al. (2020)	Yes	Yes	Offline Dataset	Precision, Recall, F-Measure Accuracy
Lilhore et al. (2020a)	No	Yes	UCI Data set	Accuracy

## 3 Materials and methods

### 3.1 Proposed methodology

This research presents a novel ensemble model that utilizes six classification models to detect DM in the Kaggle online machine repository standard dataset (PIMA Indians Diabetes dataset). This section presents the theoretical foundation and the flow of the proposed methodology for diabetes mellitus detection. As we all know, ML is the latest and upcoming area from a research point of view (Lilhore et al., 2020b).

**FIGURE 1:**
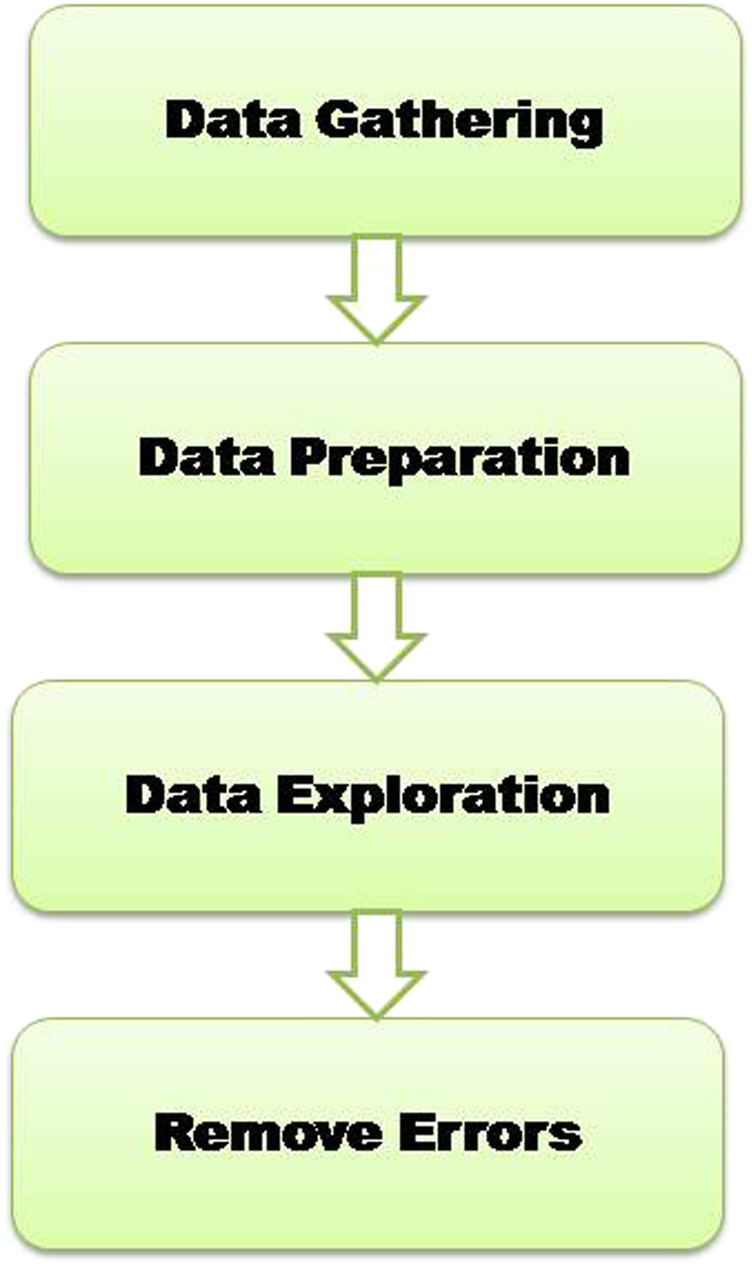
Data preparation.

Figure 1 represents the flow of consistent data agglomeration. Firstly, the dataset is used that was initially gathered from a freely available repository (Pima Indians Diabetes Database, 2022). We used the experimental dataset in the Pima Indians Diabetes database. The dataset comprises 768 distinct instances of diabetes-affected and non-affected patients. According to the dataset, all patients are female, and their age is over 21. Statistics research is the way to analyze the data and its characteristics. To clean the data, we use the data cleaning phase. When we tend the data, remove the radiance of data, and find the missing and extra values. During the cleaning phase, we observe that some attributes have 0 and missing BMI, Blood Pressure, and Glucose values. Therefore, remove these features from the dataset; there are 724 observations with nine features. A detailed description of attributes in the dataset with 0 values is given in Table 2.

**TABLE 2:** Description of attributes in the PIMA Indian Diabetes dataset that have zero values.

Name of attribute	Total number of 0 values
Blood Pressure	25
BMI	11
Glucose	5

**FIGURE 2:**
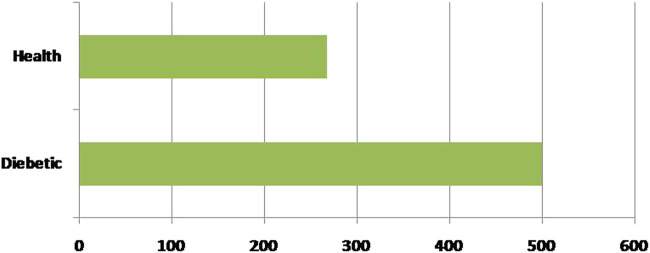
The count of healthy and unhealthy (diabetes) in the dataset.

**FIGURE 3:**
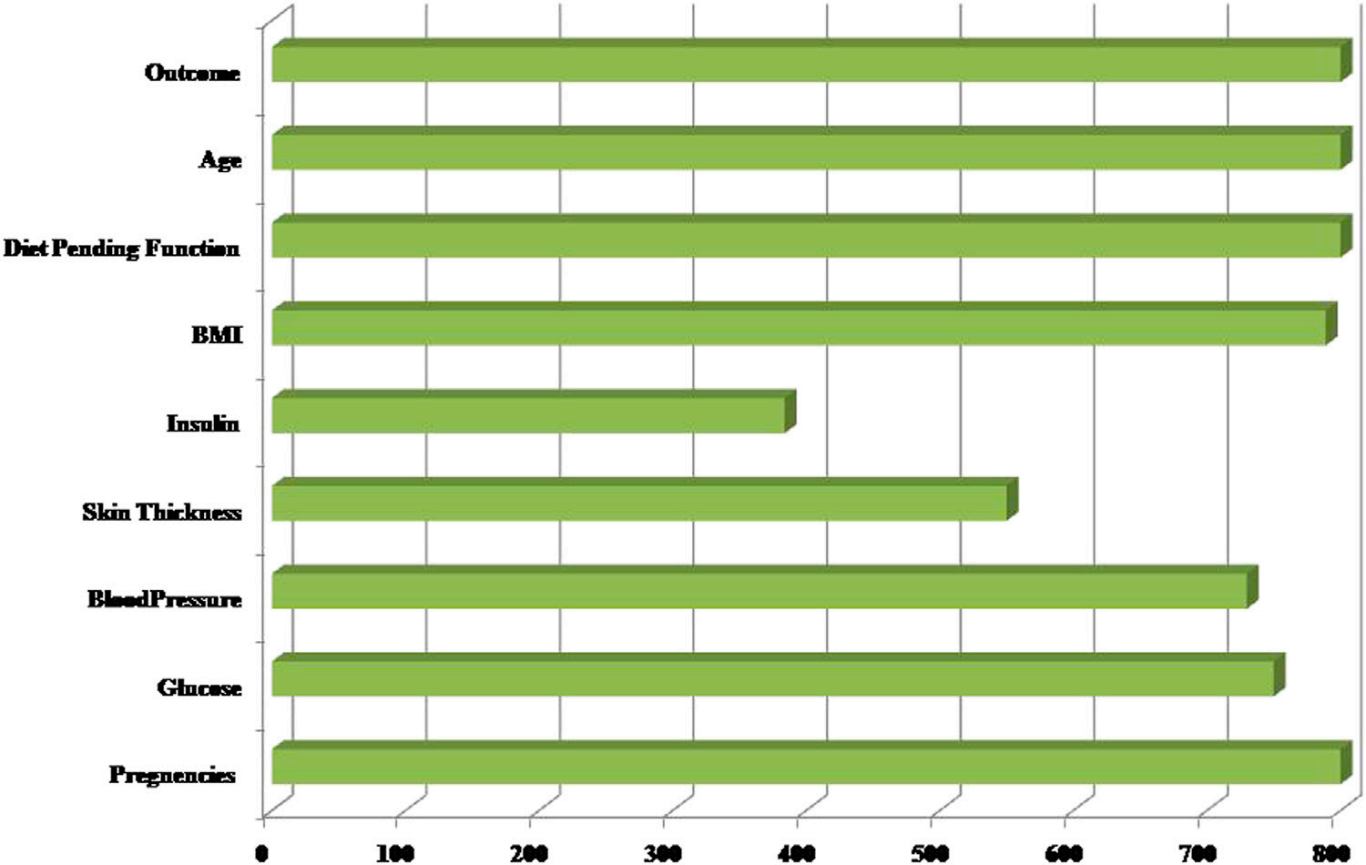
The Dataset attributes and Missing Values status.

Secondly, each feature has been measured mentioned in the dataset. Linear Discriminant Analysis (LDA) has been used as a dimensionality reduction approach to extract the subsets from the significant features (Guleria et al., 2020). It minimizes time and space complexity and enhances accuracy. The next step will train classifiers with optimum tuning parameters using the dataset. Figure 2 represents the dataset’s healthy and unhealthy (diabetes) count. Figure 3 illustrates the dataset attributes details (total of nine attributes) and Missing values status.

Figure 5 depicts the multilevel ensemble model created using a combination of models. The pseudo-code of the proposed model is shown in Figure 6. Finally, the performance of the proposed model has been evaluated on various parameters such as Recall, Precision, F-measure, and area under the ROC curve (AUC) with repeated k-fold cross-validation.

### 3.2 Preliminaries

**FIGURE 4:**
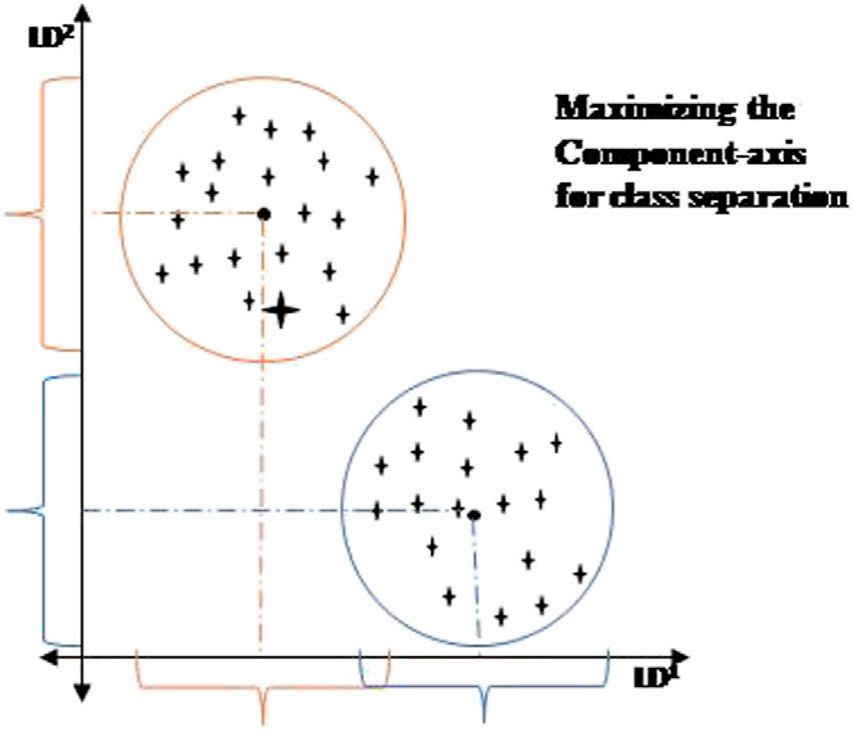
Pictorial representation of LDA.

LDA: Linear Discriminant Analysis is a technique for reducing the dimensionality of the dataset (Sharma et al., 2021a). It reduces the number of dimensions in a dataset while maintaining the maximum amount of information. LDA creates a new axis using the data from both features and projects the information onto it. It seeks to maximize the distance between the mean of each class while minimizing spreading within the class. Finally, LDA utilizes class measures. This choice provides the optimal classification results (Simaiya et al., 2021). When data has been projected in lower-dimensional space, it increases the distance between the means of every class. The pictorial representation of the LDA model is depicted in Figure 4.

The five main phases of the LDA model are:

1 Calculate the mean vectors in d dimensions for different data set classes.
2 The scatter matrices calculation (both within-class and between-class).
3 For the above calculated scatter matrices, computation of the Eigenvalues (β_1_, β_2,_ … , β_k_) and respective eigenvectors (α_1_, α _2,_ … , α _k_).
4 Now select n eigenvectors with the maximum value of Eigenvalues so that a k × n dimension matrix ѡ will be generated by lowering the order of Eigenvalues.
5 To renew the samples over the various subspaces, use this Eigen vector-matrix ѡ created above. The matrix multiplication can be presented in the short term as Z = X × ѡ.

### 3.3 Models used for ensemble learning

#### 3.3.1 SVM

The SVM is a traditional machine learning method (Hingane and Lilhore, 2018). This method is used extensively in the field of diabetes classification. The kernel function is used if the data cannot be separated linearly. Standard kernel functions include RBF, poly-function, linear, and sigmoid functions. We use linear for the SVM method as a kernel function in this research.

#### 3.3.2 KNN

The kNN method is a conventional data mining method used for many years to detect diabetes (Shrivas et al., 2017). The KNN method is based on measuring the distance between the various sample values. The main idea is that it is one of the most common classes in its most similar k samples in all samples for a new sample in the feature space. Usually, k is not bigger than a 20 (integer).

#### 3.3.3 Random Forest

RF is a decision-making method proposed by Breiman. Randomly all classification results are integrated with a voting strategy so that the highest frequency category is the outcome. This method is also used extensively in diabetes classification (Aher and Lilhore, 2020). RF is a multi-tree algorithm that incorporates the idea of integrated learning. Its fundamental unit is the decision tree, and the core is a varied range of integrated techniques of machine learning. Each decision tree is a classifier from the intuitive point of view. For an input sample, the results of N trees are N results. RF generates several decision trees, which differ significantly from the algorithm of the decision tree. Suppose RF predicts a new object based on some attributes. In that case, each RF tree yields results and a ‘vote’ of its own. Finally, the forest’s total output is the highest threshold of taxonomy.

#### 3.3.4 J-48

J48 is also called C4.8, the upgrade of which is C4.5. This technique selects a root node attribute, creates a division for each feature value possible, and splits the case into several subsets. Every subset reflects the root node branch, which recursively repeats this process on each component. The algorithm stops if all instances are classified as the same. In J48, information gain determines the nodes. J48 determines the information gained for every feature in each iteration. It selects the attribute with the most significant information gain value as the node.

#### 3.3.5 J-Rip

Repeated Incremental Pruning to Produce Error Reduction (RIPPER) is implemented by JRip, an optimized version of Incremental Reduced Error Pruning (IREP) proposed by William W. Cohen. Reduced Error Pruning (REP) is a simple and efficient technique linked with the decision-tree algorithms based on the association rules with reduced error pruning. The training data is divided into a pruning set and a growing set for constructing rules algorithms in REP. First, an initial rule set uses a heuristic method to control the ever-increasing collection (Hassan et al., 2021). This overriding rule set is further simplified repetitively by applying an atypical pruning operator to remove any single rule or condition.

#### 3.2.6 Naive Bayes

The Naïve Bayes classification process is a probabilistic Bayes theorem-based learning technique (Sharma et al., 2021b). It outperforms other classifications, even in its simplicity, and is thus better performing classification algorithms. The Bayes theorem is presented below for calculating the posterior probability:

$$⍴\left(r|s\right)=\frac{⍴\left(s|r\right).p\left(r\right)}{⍴\left(s\right)}$$ (1)

**FIGURE 5:**
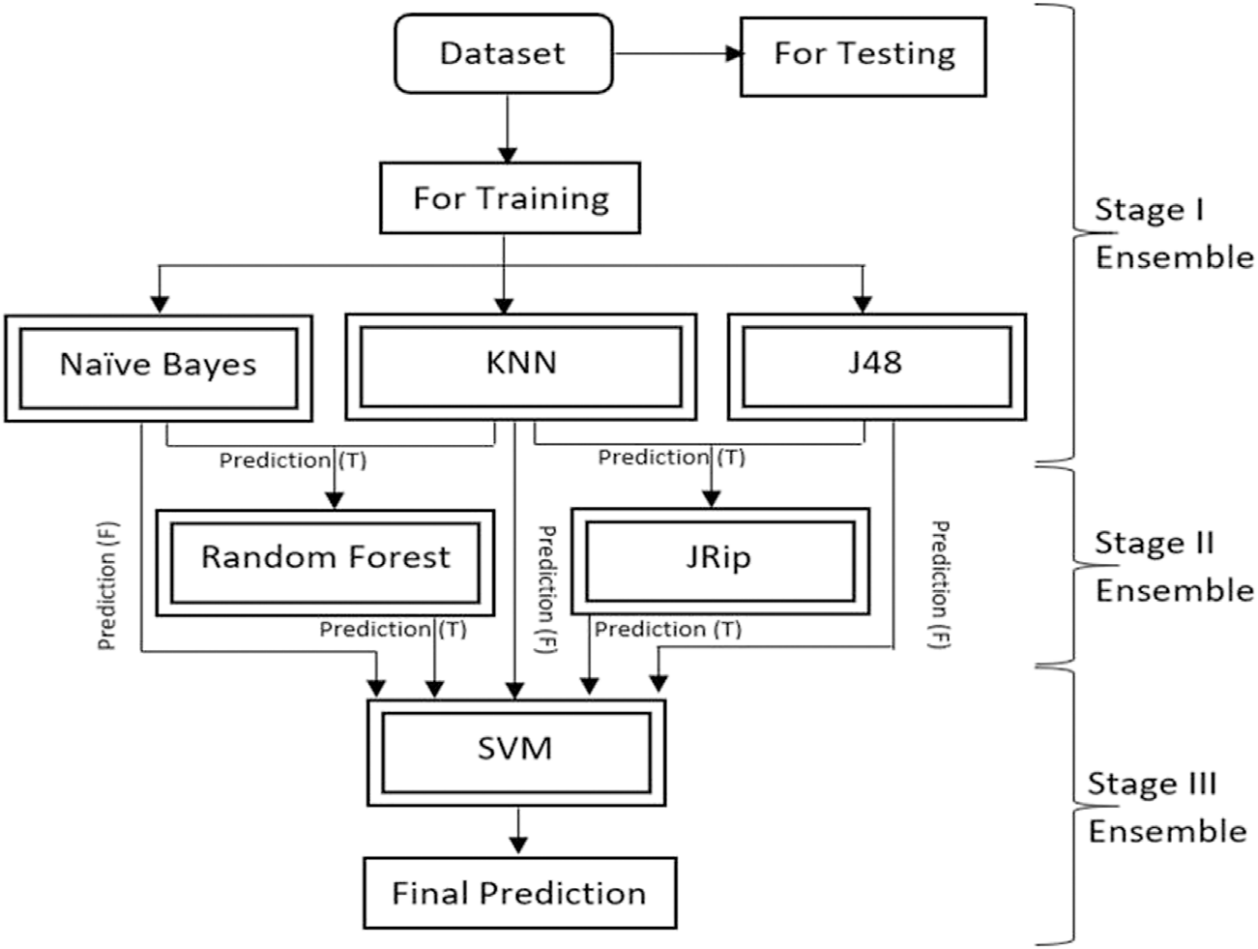
The proposed ensemble approach.

**FIGURE 6:**
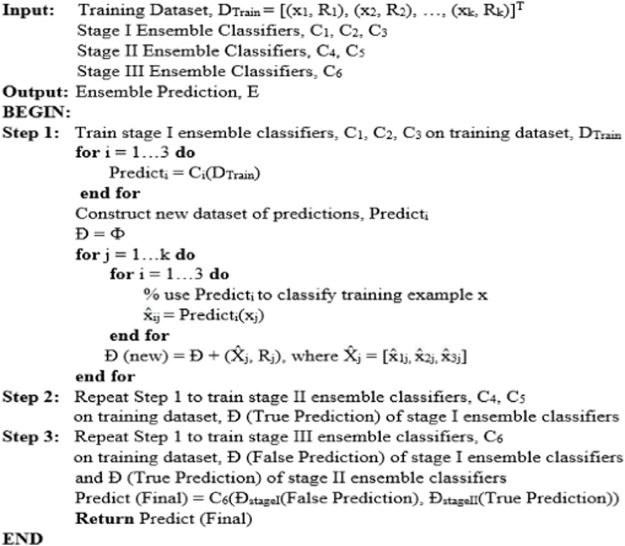
Pseudo code of the Proposed Ensemble Approach.

Where, $⍴\left(rs\right)$ is Posterior Probability, $⍴\left(s\right)$ is Predictor Prior Probability, $p\left(r\right)$ is Class Prior Probability, and $⍴\left(sr\right)$ is Likelihood. As shown in Figure 3, a schematic diagram demonstrates the complete process for the proposed ensemble model. The model phase begins with the input dataset reading. Various preprocessing operations are then implemented, such as finding the extra and missing values and removing the data’s radiance. The LDA method as the extraction technique is applied in the next step. The next phase involves utilizing six existing machine-learning models to create an ensemble model that outperforms all of these models. The ensemble method handles the worst-case scenario of model prediction (Khan et al., 2022). The current study focuses on the model’s true and false predictions. A multilevel ensemble model is employed to cope with both true and false predictions. There are six models in total, i.e., As shown in Figure 5, SVM, KNN, Random Forest, J48, JRip, and Naive Bayes are coupled to improve accuracy. These models are tested on 30% of the dataset, with 70% used for training. Figure 6 shows the detailed pseudo code of the proposed model. Pseudo-code represents the structure and sequence of steps we use to detect DM.

The ensemble model is separated into three stages, each of which is described in detail below:

• Stage I: 70% of the dataset is used to train the Naive Bayes, KNN, and J48 models, and 30% is used to create predictions.
• Stage II: The RF model trained the data using the true predictions of two models (Naive Bayes and KNN) from stage I. Another J-Rip model trained the data using the true predictions of two models (KNN and JRip) from stage I.
• Stage III: The true stage II predictions and the false stage I predictions are integrated. The SVM model generates final predictions, which are trained using this combined new dataset. True and false predictions are refined in the current methodology to produce an effective model. The data is passed through six models, which allow the models to learn the data precisely and produce accurate and reliable results. The use of true prediction as an input to other models is intended to prevent false-positive findings (Non-diabetic is considered as diabetic).

## 4 Results and discussions

In the current work, the proposed ensemble model is implemented in Python using Scikit-Learn. The proposed model is based on a recursive feature elimination approach to finding the potential features and improving the accuracy. Table 2 represents the performance of various ML models and the proposed model to detect diabetes. In this research, we analyze the performance of existing ML techniques and compare the analytics output with the proposed method. The current approaches consider the entire feature space for computing the accuracy.

### 4.1 Performance evaluation

There are different parameters for assessing models, such as Recall, Precision, f-measure, and AUC; all these parameters are utilized in this study to evaluate the proposed model.

#### 4.1.1 Recall

It is also referred to as true positive rate or sensitivity (Eq. 2) (Yang et al., 2021). It entails the proportion of true positive elements that are correctly identified by the classifier and calculated as:

$$Recall=\frac{TP}{TP+FN}$$ (2)

#### 4.1.2 Precision

It is also known as positive predictive value (Eq. 3) (Zhu et al., 2015). It relates to the ability of the classifier to identify the fraction of positive results to all returned results:

$$Precision=\frac{TP}{TP+FP}$$ (3)

#### 4.1.3 F-Measure

It is computed from precision and recall (Eq. 4) (Zou et al., 2018). The maximum possible value for an F-Measure is 1.0, indicating perfect accuracy and Recall, and the minimum possible value is 0. It is the harmonic mean of Precision and Recall as:

$$F-Measure=2\times \frac{Precision\times Recall}{Precision+Recall}$$ (4)

#### 4.1.4 Confusion Matrix

**FIGURE 7:**
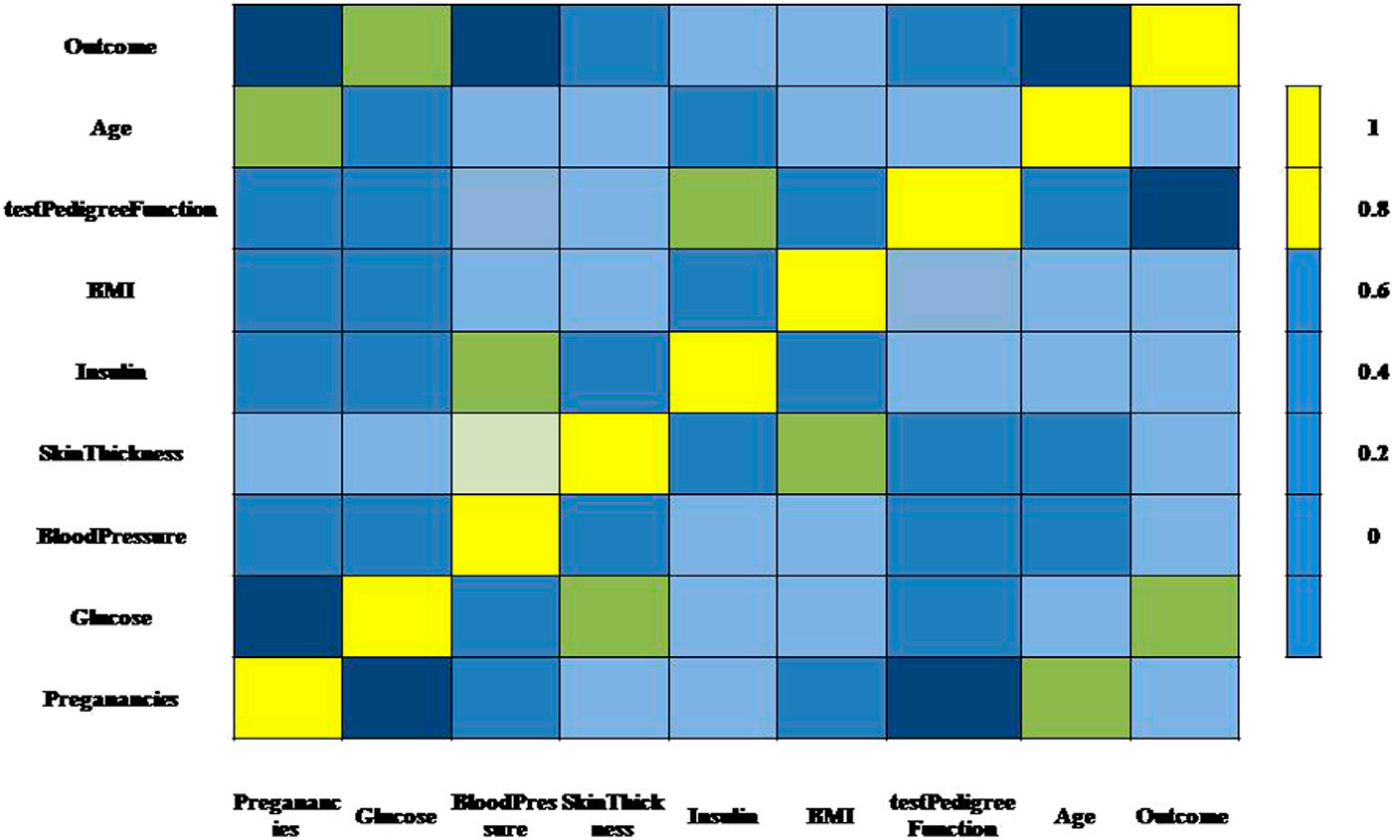
Confusion Matrix for the proposed model.

This table is frequently used to define the model’s accuracy (“classifier”) on just various experimental test data values computed. Significant predictor analyses such as Precision, Accuracy, Recall, and specificity are illustrated using a confusion matrix. Confusion matrices have been necessary because they enable you to directly compare false positives, False Negatives, True Negatives, and true positives. Figure 7 represents the confusion matrix for the proposed model.

#### 4.1.5 Area under the curve

AUC (Area under the curve) measures the quality of a classification model. The area of the ROC (receiver operating characteristics) curve is AUC. The high AUC value of the model is considered an effective model compared to other approaches. It has a value from 0 to 1. Model performance is better if the value of AUC is close to 1 (Trivedi et al., 2020).

### 4.2 Repeated K-Fold cross-validation

A large number of observations are always recommended to compare the model’s performance. Repeated K-cross validation (Lilhore et al., 2020a) helps carry out K-fold cross-validation numerous times or increase comparative analyses. Only k comparisons are acquired in K-fold cross-validation. Random data is available for comparisons in each fold for cross-validation. In the current work, 10-fold cross-validation is repeated three times. The overall performance of the proposed model is described in Table 3. The performance was determined by performing three rounds of 10-fold cross-validation. Figure 12 illustrates the effectiveness of the proposed method three times in ten runs. It demonstrates the consistency in the classification accuracy of the proposed method using a box plot.

**TABLE 3:** Ensemble approach result table.

Parameters	SVM	Naïve bayes	JRip	J48	RF	KNN	Proposed ensemble model
Recall	0.762	0.743	0.760	0.738	0.758	0.754	**0.786**
Precision	0.757	0.741	0.755	0.735	0.754	0.750	**0.784**
F-Measure	0.759	0.742	0.757	0.736	0.756	0.752	**0.785**
AUC	0.816	0.806	0.739	0.751	0.820	0.793	**0.854**

The bold values show the results for Proposed Method.

The models could also become biased during training; the SMOTE (Lilhore et al., 2020b) method is used in the current work to address this issue. Other problems include underfitting/overfitting and if performance is consistent, i.e., models are underfitting/overfit free. The model should be tested and cross-validated on an independent dataset. Underfitting means learning too little, and overfit model knows too much. Models are executed n times during cross-validation, and their accuracy is measured; if Precision fluctuates wildly, the model is underfitting/overfit/biased.

Another problem is that to deal with the overfitting/underfitting issue, the model should be cross-validated and tested on an independent dataset; if performance is consistent, then models are free from overfitting/underfitting. Overfitting is when models learn too much, and underfitting is when models know too little. In cross-validation, models are executed n times, and accuracy is recorded if accuracy is highly fluctuating. That model is overfitted/under fitted/biased. Repetitive K-fold cross-validation describing consistency inaccuracy is used in current work, implying that these problems do not impact the proposed model. The proposed model is free of biased, underfit and overfit issues in the recent work and is improved as a result of the proposed model compared with the literature work (AlGarni et al., 2022; Ul Hassan et al., 2022).

**FIGURE 8:**
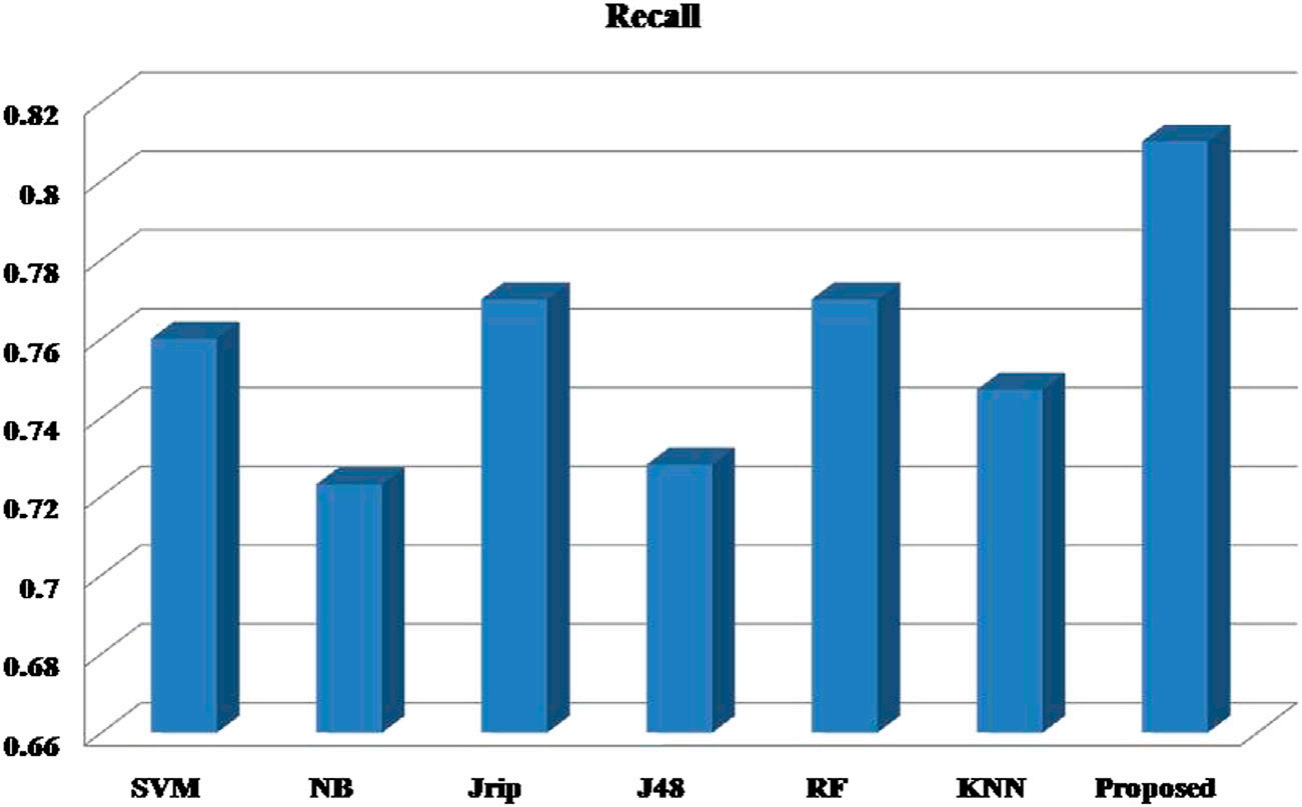
Experimental results for Recall.

**FIGURE 9:**
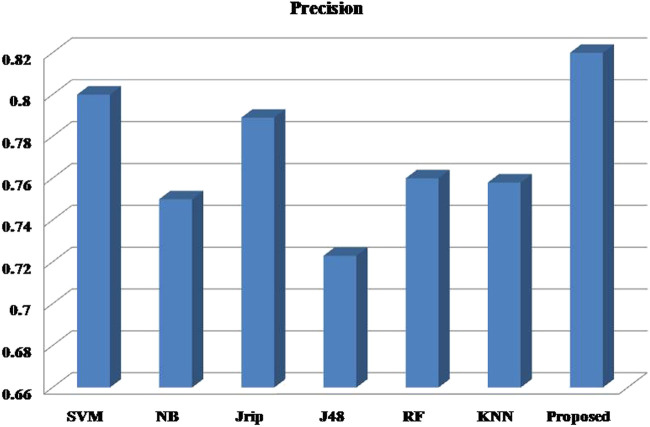
Experimental results for Precision.

**FIGURE 10:**
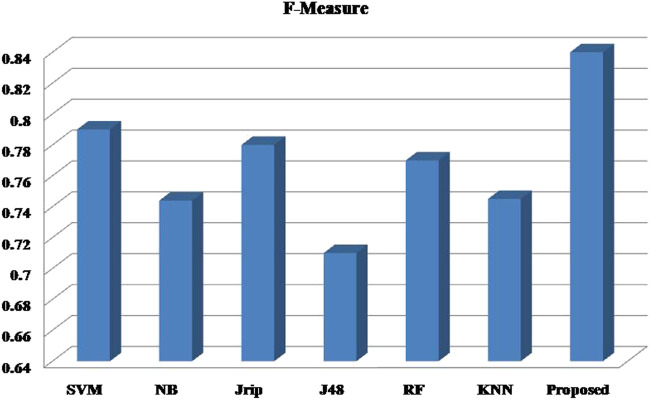
Experimental results for F-Measure.

**FIGURE 11:**
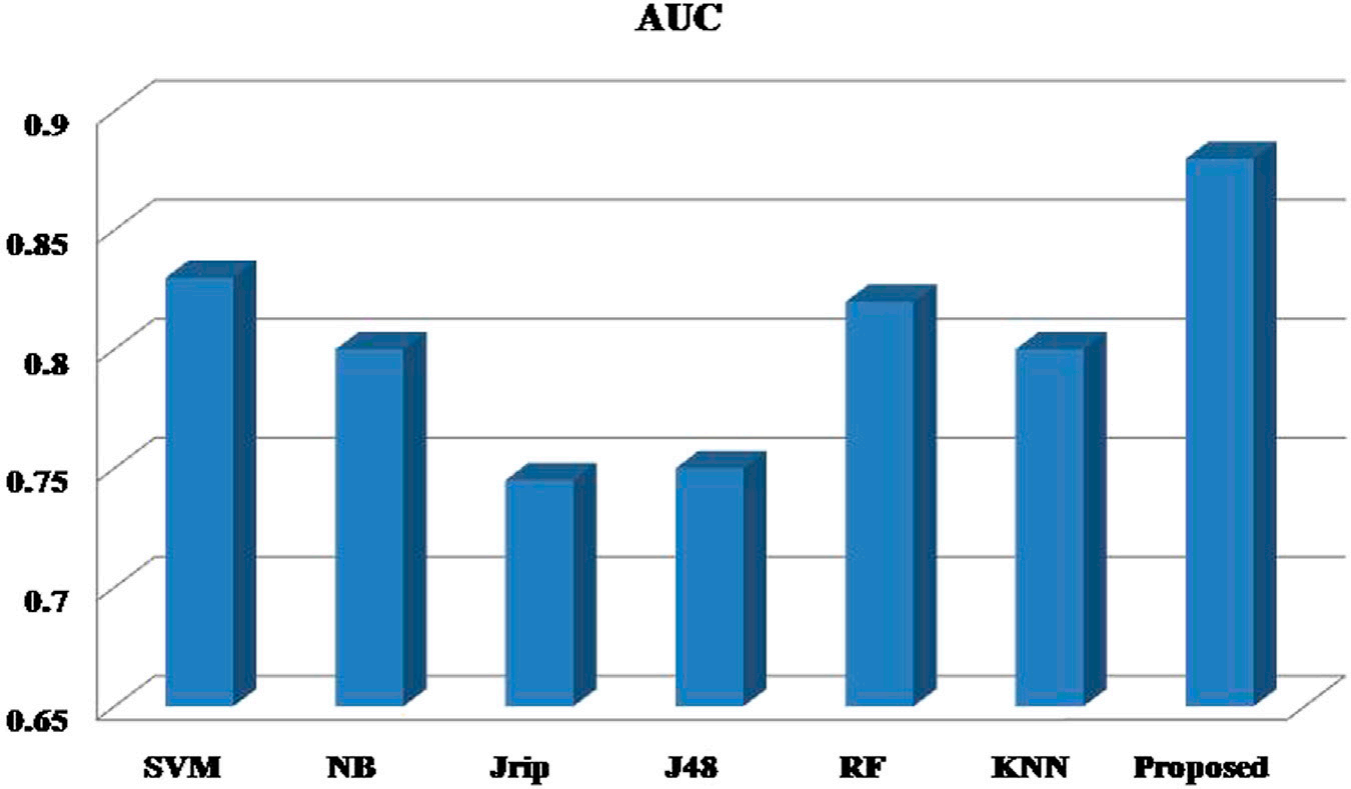
Experimental results for AUC values.

Six machine learning models are utilized in experiments for the proposed ensemble model. Table 2 provides average Recall, Precision, and f-measure together with Area Under ROC Curve (AUC) values for ten runs (Maximum values are depicted as bold). Table 3 showcases that in terms of all evaluation metrics, the proposed model surpasses other models. The boxplots for six machine learning models and the proposed ensemble model are shown in Figure 8, 9, 10, and 11. The *x*-axis in all these (Figure 8, 9, 10, and 11) represents the model names. In contrast, the *y*-axis denotes the parameter under consideration, i.e. (Recall, Precision, f-measure, and AUC).

**FIGURE 12:**
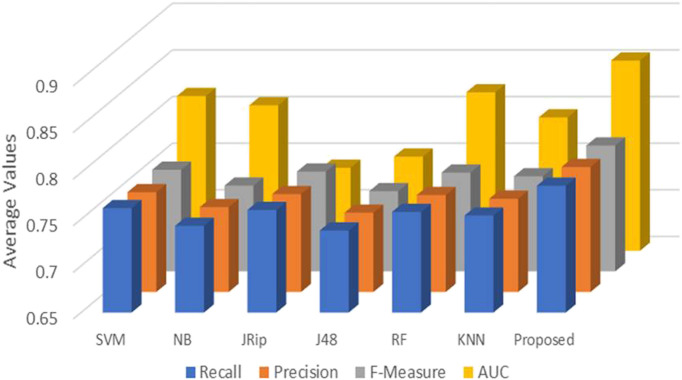
Average Recall, Precision, F-Measure, and AUC values.

Figure 12 compares the proposed model to other models using various performance measures such as average f-measure, Precision, Recall, and AUC. The CS-SVM model does have significantly higher Recall, Precision, and f-measure values for predicting diabetes patients.

## 5 Conclusion and future work

Diabetes is a metabolic disorder that influences people all over India and other countries. World Health Organization gives the dataset showing that frequency increases significantly every year. Diabetes can be fatal to any vital part of the body if left untreated. Firstly, it identified the DM to provide the appropriate treatments. Suppose a person can ignore diabetes, which causes a significant problem for the human body. So to get better health and proper treatments must detect diabetes at an early stage, which can prevent the condition from escalating to severe problems; in the medical field challenging to achieve the accuracy of the dataset. ML approaches are used nowadays to detect diabetes. The proliferation of electronic data has led to the development of complex predictive models that can be updated using machine learning models. This paper is the key to finding the best formulation for a diabetes prognosis.

We emphasized covering this using an ensemble approach for predicting diabetes. The main contribution of the proposed system is the development of a novel method for diabetes detection, even in the presence of a large number of lesions, and the use of hybrid approaches to enhance the accuracy of the machine learning model. The evaluation is done using the Pima Indian dataset. SVM, Naïve Bayes, J Rip, J48, KNN, and RF make an ensemble approach. Results and accuracy are shown in different tables. The model is trained using a dataset, and results demonstrate greater effectiveness over existing models. In future research, we aim to integrate its Smartphone application for the proposed hypothetical pre-diabetes tracking system with the envisaged pattern classification strategies. Genetic programming and neural networks can all be combined with the proposed solution framework for improved observation in an actual environment.

## Data Availability

The original contributions presented in the study are included in the article/Supplementary Material, further inquiries can be directed to the corresponding authors.
